# Adjuvant Therapy with Oncolytic Adenovirus Delta-24-RGDOX After Intratumoral Adoptive T-cell Therapy Promotes Antigen Spread to Sustain Systemic Antitumor Immunity

**DOI:** 10.1158/2767-9764.CRC-23-0054

**Published:** 2023-06-27

**Authors:** Hong Jiang, Dong Ho Shin, Yanhua Yi, Xuejun Fan, Joy Gumin, Jiasen He, Andrew G. Gillard, Frederick F. Lang, Candelaria Gomez-Manzano, Juan Fueyo

**Affiliations:** 1 Department of Neuro-Oncology, The University of Texas MD Anderson Cancer Center, Houston, Texas.; 2 Department of Neuro-Surgery, The University of Texas MD Anderson Cancer Center, Houston, Texas.; 3 Pediatric division, The University of Texas MD Anderson Cancer Center, Houston, Texas.

## Abstract

Cancer cell heterogeneity and immunosuppressive tumor microenvironment (TME) pose a challenge in treating solid tumors with adoptive cell therapies targeting limited tumor-associated antigens (TAA), such as chimeric antigen receptor T-cell therapy. We hypothesize that oncolytic adenovirus Delta-24-RGDOX activates the TME and promote antigen spread to potentiate the abscopal effect of adoptive TAA-targeting T cells in localized intratumoral treatment. Herein, we used C57BL/6 mouse models with disseminated tumors derived from B16 melanoma cell lines to assess therapeutic effects and antitumor immunity. gp100-specific pmel-1 or ovalbumin (OVA)-specific OT-I T cells were injected into the first subcutaneous tumor, followed by three injections of Delta-24-RGDOX. We found TAA-targeting T cells injected into one subcutaneous tumor showed tumor tropism. Delta-24-RGDOX sustained the systemic tumor regression mediated by the T cells, leading to improved survival rate. Further analysis revealed that, in mice with disseminated B16-OVA tumors, Delta-24-RGDOX increased CD8^+^ leukocyte density within treated and untreated tumors. Importantly, Delta-24-RGDOX significantly reduced the immunosuppression of endogenous OVA-specific CTLs while increasing that of CD8^+^ leukocytes and, to a lesser extent, adoptive pmel-1 T cells. Consequently, Delta-24-RGDOX drastically increased the density of the OVA-specific CTLs in both tumors, and the combination synergistically enhanced the effect. Consistently, the splenocytes from the combination group showed a significantly stronger response against other TAAs (OVA and TRP2) than gp100, resulted in higher activity against tumor cells. Therefore, our data demonstrate that, as an adjuvant therapy followed TAA-targeting T cells in localized treatment, Delta-24-RGDOX activates TME and promotes antigen spread, leading to efficacious systemic antitumor immunity to overcome tumor relapse.

**Significance:**

Adjuvant therapy with oncolytic viruses promotes antigen spread to potentiate localized intratumoral adoptive T-cell therapy with limited TAA targets, leading to sustainable systemic antitumor immunity to overcome tumor relapse.

## Introduction

Despite years of advances in cancer therapy, metastasis remains devastating and accounts for more than 90% of cancer-related deaths (1). Chimeric antigen receptor (CAR) T-cell therapy and oncolytic virotherapy are two biological therapies being tested clinically to treat advanced metastatic solid tumors. Unlike its remarkable success in treating hematologic malignancies, the effect of CAR T-cell therapy is modest in solid tumors (2). In part, this suboptimal efficacy can be attributed to poor infiltration and inactivation of T cells in solid tumors with an immune-suppressive tumor microenvironment (TME; “cold tumor”; ref. 3). Moreover, due to limited available targets for CAR T cells and the heterogeneity of solid tumors, the therapy may spare the cancer cells with loss or downregulation of the target antigens, referred to as antigen escape, leading to tumor relapse (4, 5). One potential approach to increase tumor access is to perform regional delivery. To this end, CAR T cells are infused intraventricularly or intracerebroventricularly to treat patients with gliomas (6, 7). CAR T cells are well tolerated and evoke clinical and radiographic improvement (6, 7). However, it is unclear whether localized intratumoral delivery of CAR T cells will mediate an abscopal effect.

Oncolytic viruses (OV) are genetically modified or naturally occurring viruses that selectively replicate in and disrupt cancer cells (8). They are emerging as promising immunotherapy drugs for cancer. In 2015, Amgen's T-Vec (talimogene laherparepvec) became the first OV approved by the FDA to treat surgically unresectable skin and lymph node lesions in patients with metastatic melanoma (9). To this end, in a phase I clinical trial with Delta-24-RGD, a second-generation oncolytic adenovirus (OA) derived from human adenovirus serum type 5 targeting tumor cells with an aberrant RB pathway (10–12), we observed that intratumoral injection of the virus induced an inflammatory response and a durable complete response in a subset of patients with recurrent glioblastoma (13). To further increase the efficacy and specificity of the virus, we constructed the next-generation OA Delta-24-RGDOX that expresses immune costimulatory OX40 ligand (OX40L). Compared with its predecessor, this new virus elicits more potent *in situ* autologous cancer vaccination, resulting in efficacious, tumor-specific, and long-lasting therapeutic effects in immunocompetent mouse glioma models (14). Furthermore, in melanoma mouse models with disseminated subcutaneous and intracranial tumors, Delta-24-RGDOX induces systemic antitumor immunity when it is injected into the primary melanoma (15). DXN-2440, a human version of Delta-24-RGDOX, is being investigated clinically for patients with recurrent glioblastoma (NCT03714334, ClinicalTrials.gov) and colorectal cancer and other tumors with liver metastases (NCT04714983). Nevertheless, previous clinical trials involving oncolytic virotherapy revealed that only a small portion of the patients (<10%) demonstrated an overall objective response (16). Thus, combination therapies are necessary to realize the full potential of this treatment strategy.

Preclinical and clinical studies have suggested that the aforementioned approaches are complementary (17–19). During oncolytic virotherapy, we and others observed an increased presence of T cells in the virus-injected tumor (13–15, 20, 21), turning a “cold tumor” into an immunogenic “hot tumor.” Moreover, immune activation extends to disseminated untreated tumors and peripheral lymphoid organs (15, 21). Importantly, Delta-24-RGD and its derivatives have the potential to lyse the whole cancer cell population and mediate the presentation of the entire cancer antigen repertoire to the immune system to instigate an *in situ* autovaccination favorable for the proliferation of all tumor-targeting T-cell populations. Thus, it is possible to overcome the resistance of cancers due to their heterogeneity and therapy-induced evolution of the tumor cells, which are the main challenges in developing targeted cancer therapies. On the other hand, because it takes time to develop virus-mediated antitumor immunity, it is challenging for the virus to control fast-growing large tumors in a timely manner. The instant potent action of an adequate number of tumor-targeting T cells should compensate for viral anticancer activity at this stage. Thus, we hypothesize that Delta-24-RGDOX activates TME and complements the limitation of TAA targets during adoptive T-cell therapy in localized intratumoral treatment, instigating *in situ* autovaccination and leading to efficacious and sustainable systemic antitumor effect. To test this hypothesis, we combined TAA-targeting CTLs derived from transgenic mice and Delta-24-RGDOX in the localized intratumoral treatment of immunocompetent mice with disseminated tumors. Because these CTLs express the T-cell receptor (TCR) targeting a single TAA, we think this experimental setting can be used to study the antigen escape problem encountered by adoptive T-cell therapy targeting a single TAA, such as CAR T-cell therapy. We characterized the immune response during the treatments to dissect the mechanism underlying the antitumor effect induced by the combination

## Materials and Methods

### Cells

The mouse melanoma cell lines B16F10 Red-FLuc-3 and B16-ovalbumin (OVA; ref. 15) were grown in RPMI1640 medium supplemented with 10% FBS (HyClone Laboratories, Inc.), 100 μg/mL penicillin, and 100 μg/mL streptomycin. Human lung carcinoma A549 cells (ATCC) were cultured in DMEM nutrient mixture F12 (DMEM/F12) supplemented with 10% FBS and antibiotics. Human embryonic kidney 293 cells (QBioGene, Inc.) and mouse lung carcinoma CMT64 cells (Culture Collections, Public Health England) were maintained in DMEM supplemented with 10% FBS and antibiotics. All cells were kept at 37°C in a humidified atmosphere containing 5% CO_2_. Experiments were conducted within 6 months after the cell lines were obtained from a cell bank or within 15 passages in the laboratory. All cell lines were tested and found to be free of *Mycoplasma*.

### Viruses

The Delta-24-RGDOX viruses were propagated in A549 cells and titrated in 293 cells as described previously (14).

### Mice

MD Anderson's Mouse Resource Facility provided the C57BL/6 mice. OT-I [C57BL/6-Tg(TcraTcrb)1100Mjb/J, stock no: 003831] and pmel-1 [B6. Cg-*Thy1^a^*/Cy Tg(TcraTcrb)8Rest/J, stock no: 005023] breeding pairs were purchased from The Jackson Laboratory and then bred at MD Anderson's animal facility.

### Preparation of Splenocytes and CD8^+^ T-cell Isolation and Expansion

Mouse spleens were collected and processed as described previously (14). CD8^+^ T cells were enriched from the splenocytes with a mouse CD8a^+^ T-Cell Isolation Kit (Miltenyi Biotec, Inc.). Then, the cells were expanded as described previously with modifications (22). Briefly, the cells were activated in 6-well plates (5 × 10^6^ cells/5 mL/well) coated with anti-CD3 (0.5 μg/mL, clone 2C11, Bio X Cell) and anti-CD28 (5 μg/mL, clone 37.51, Bio X Cell) for 20 hours in RPMI1640 medium containing 10% FBS and antibiotics and supplemented with 2 mmol/L L-glutamine, 50 μmol/L 2-mercaptoethanol, 1% insulin-transferrin-selenium (ITS-G, Thermo Fisher Scientific), 6 ng/mL mouse IL2 and 0.5 ng/mL mouse IL7 (ProSpec). After another 48 hours expansion, the cells were harvested and used for adoptive T-cell therapy in mice.

### Animal Studies

Six- to 10-week-old mice were used for tumor therapeutic studies. To set up disseminated tumors in the mice, on day 0, B16F10 Red-FLuc-3 or B16-OVA cells (5 × 10^5^ cells/mouse) were injected subcutaneously (s.c.) into the lower back of the mice. On day 4, the same cells (1 × 10^6^ cells/mouse) were injected subcutaneously approximately one inch away from the first tumor on the back close to the neck and/or grafted into the caudate nucleus of the mouse brain (B16F10 Red-Fluc-3, 2,000 cells/mouse) using a guide-screw system as described previously (11). On the same day that the T-cell treatment was initiated, the mice with implanted tumors were evaluated first with bioluminescent imaging (15) or tumor volume measurement (*V* = 0.5 × *L* × *W* × *W*) to assess tumor growth. Mice with off-size tumors were eliminated from the study, and the rest of the mice were randomly assigned to experimental groups. CTLs (OT-I T cells: 1 × 10^6^ cells/mouse; pmel-1 T cells: 3 × 10^6^ cells/mouse) were injected into the first implanted subcutaneously tumor, followed by three doses of Delta-24-RGDOX (2 × 10^8^ plaque-forming units/mouse). To rechallenge the surviving mice, B16-OVA (5 × 10^5^ cells/mouse) or CMT64 (1 × 10^6^ cells/mouse) cells were implanted subcutaneously into the backs of the mice. Tumor volume was measured three times a week. For the survival study, mice were euthanized when the sum of the longer diameters of the two subcutaneous tumors was ≥ 2 cm. Mice with ulceration more than 5 mm diameter were eliminated from the studies. All animal studies were conducted in C57BL/6 mice. All experimental procedures involving the use of mice were performed in accordance with protocols approved by MD Anderson's Animal Care and Use Committee and followed U.S. NIH and Department of Agriculture guidelines. We used the ARRIVE Guidelines for reporting animal research (23).

### Immunoblotting

The cells were collected and resuspended in PBS plus protease inhibitor cocktail (Sigma-Aldrich) and then subjected to lysis by adding an equal volume of 2 × sodium dodecyl sulfate loading buffer. Then the lysates were heated at 95°C for 10 minutes. Equal amounts of proteins from the lysates were separated by SDS-PAGE, transferred to a nitrocellulose membrane, and probed with antibodies. Finally, the protein bands were visualized using an enhanced chemiluminescence Western blot detection system (Amersham Pharmacia Biotech) and quantified with ImageJ (https://imagej.nih.gov).

### Enrichment of Leukocytes from Tumors

The leukocytes from intacranial and subcutaneous tumors were enriched via centrifugation through Percoll gradient medium (GE Healthcare Bio-Sciences) as described previously (14, 15).

### Flow Cytometry Analysis

To analyze cell surface protein expression, we blocked the cells (up to 1 × 10^6^) with CD16/32 (10 μg/mL) and then incubated them in 100 μL of primary antibody solution diluted in PBS plus 3% BSA and 1 mmol/L ethylenediaminetetraacetic acid. After incubation at 4°C in the dark for 30 minutes, the cells were washed once with 1 mL of cold PBS. Then, the cells were resuspended in 0.5 mL of PBS. The stained cells were analyzed using flow cytometry (BD FACSCelesta Cell Analyzer). The antibodies and tetramers used for staining the cells are listed in Supplementary Table S1. Cell counts were obtained using 123 count eBeads (01-1234, eBioscience) as a control for calculating processed sample volumes that were used to quantify the cell density within the tumors. Dead cells were excluded from the analysis through staining with Ghost Dye Violet 510 (13-0870, Tonbo Biosciences).

### Stimulation of Splenocytes

To prepare the target cells, 50 units/mL mouse IFNγ (Prospec Protein Specialists) was added to the B16-OVA cell culture. Forty-eight hours later, the cells were fixed with 1% paraformaldehyde and washed before being added to the coculture. For immune cell stimulation, splenocytes (5 × 10^5^ cells/well) were incubated with prefixed target cells (B16-OVA, 5 × 10^4^ cells/well) or one of the following peptides at 2 μg/mL: mgp100 (refs. 24–32; AnaSpec), OVA (257–264; Invitrogen), TRP-2 (180–188; AnaSpec), or GFP (118–126; Biomatik). Twenty-four hours after incubation in a round-bottom 96-well plate, the concentration of IFNγ in the medium was assessed with a Mouse IFNγ DuoSet ELISA kit (R&D Systems).

### Statistical Analysis

In quantitative studies of cultured cells and flow cytometry, each group consisted of triplicate samples. Each study was repeated at least once. Differences between groups were evaluated using a two-tailed Student *t* test. The animal survival curves were plotted according to the Kaplan–Meier method, and *P* values were computed with the log-rank test using GraphPad Prism software. *P* values < 0.05 were considered significant, shown as asterisks (*, *P* < 0.05; **, *P* < 0.01; ***, *P* < 0.001; ****, *P* < 0.0001). The coefficient of drug interaction (CDI) was used to analyze effects of T cells and Delta-24-RGDOX combination. CDI is calculated as follows: CDI = *AB*/(*A* × *B*). According to the effect of each group, *AB* is the ratio of the combination group to control group; *A* or *B* is the ratio of the single agent group to control group. Thus, CDI <1, = 1, or >1 indicates that the drugs are synergistic, additive, or antagonistic, respectively. CDI < 0.7 indicates that the drugs are significantly synergistic (33).

### Data Availability

The data generated in this study are available upon request from the corresponding author.

## Results

### Localized Intratumorally Injected TAA-targeting T cells Show Tropism for Distant Disseminated Tumors and Tumor-draining Lymph Nodes

**FIGURE 1:**
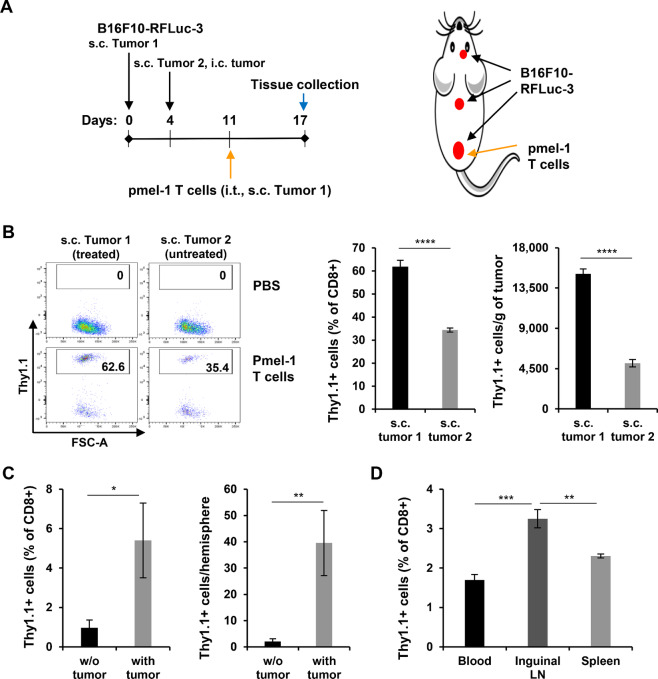
Tumor tropism of TAA-specific T cells after localized intratumoral injection. **A,** A cartoon depiction of the treatment scheme for schedule (left) and positions of tumor implantation and T-cell injection (right). s.c.: subcutaneously; i.t.: intratumorally; i.c.: intacranially. **B–D,** Flow cytometry profiling of leukocytes from the indicated tissues from the treatment groups to show the distribution of pmel-1 T cells (Thy1.1+) in the subcutaneous tumors (**B**, left: representative flow cytometry dot plots; right: quantification of Thy1.1+ cells in the tumors), brain hemispheres (**C**) and peripheral lymphoid organs (**D**). The numbers in the upright corner of the dot plots indicate the frequency of Thy1.1+ cells (% of CD8^+^ leukocytes). Leukocytes were from grouped tissue (PBS: *n* = 3; pmel-1 T cells: *n* = 5) and processed in triplicate. The columns in the graphs represent the mean ± SD (*n* = 3). w/o tumor: without tumor; inguinal LN: inguinal lymph node; *, *P* < 0.05; **, *P* < 0.01; ***, *P* < 0.001; ****, *P* < 0.0001. Two-tailed Student *t* test.

Regional or intratumoral delivery of CAR T cells showed a better safety profile and was promising for mediating a beneficial inflammatory response within tumors (6, 7, 24). To test whether tumor-targeting T cells injected into one tumor will show an abscopal effect, we first set up disseminated tumors in C57BL/6 mice with the mouse melanoma cell line B16F10 Red-FLuc-3 (15), which expresses melanoma-associated antigen gp100 (Supplementary Fig. S1; Fig. 1A). Then, we injected into the first established subcutaneous tumor with pmel-1 CD8^+^ T cells (CTLs, Thy1.1+), which target the gp100 epitope presented by the tumor cells (Supplementary Fig. S2A), isolated from pmel-1 mice and expanded in culture. Six days later, the biodistribution of Thy1.1+ cells was assessed with flow cytometry profiling of leukocytes from the tumors and lymphoid organs. In the subcutaneous tumors, Thy1.1+ cells made up approximately 62% of the CD8^+^ leukocytes from the treated first tumor (Tumor 1), while their proportion in the untreated distant second tumor (Tumor 2) was approximately 34% (Fig. 1B, left two panels). The density of Thy1.1+ cells in Tumor 1 was approximately 15,000 cells/g of tumor, and that in Tumor 2 was approximately 5,100 cells/g of tumor (Fig. 1B, right). Although the frequency and density of Thy1.1+ cells were much lower in the brain hemisphere with tumor than in subcutaneous tumors, these values were significantly higher than those in the lateral brain hemisphere with no tumor (Fig. 1C). In addition, the frequency of Thy1.1+ cells in inguinal lymph nodes, which are the tumor-draining lymph nodes (TDLN) for subcutaneous tumors, was significantly higher than that in blood or spleens (Fig. 1D). Collectively, the data indicate that TAA-targeting T cells injected into one tumor preferentially migrate to distant untreated tumors and TDLNs, displaying tumor tropism.

### Localized Intratumoral Treatment with TAA-targeting T cells Followed by Delta-24-RGDOX Induces Sustainable Systemic Tumor Regression

**FIGURE 2:**
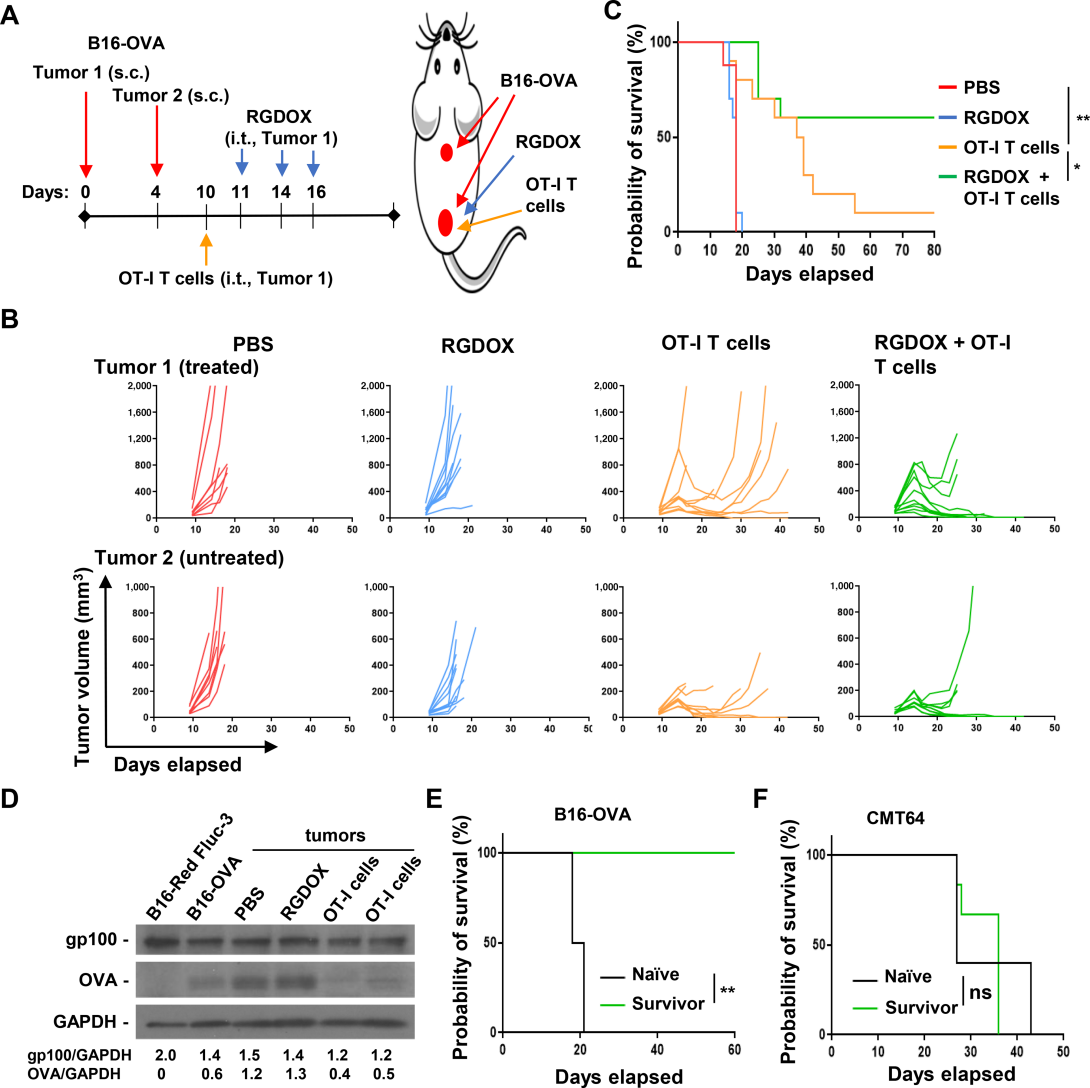
Systemic tumor regression sustained by combining TAA-specific T cells and Delta-24-RGDOX in localized intratumoral treatment. **A,** A cartoon depiction of the treatment scheme for schedule (left) and positions of tumor implantation, T-cell and virus injection (right). s.c.: subcutaneously; i.t.: intratumorally. **B,** Spaghetti plots of the volume of the tumors from the indicated treatment groups. **C,** Survival plots of the treatment groups. *n* = 10 except *n* = 8 for PBS. **D,** The cell lysates from the indicated cell lines or tumors were analyzed with immunoblotting for OVA (the target of OT-I cells) or gp100. Note that the tumors from PBS or RGDOX group were treated ones collected 8 days after OT-I cell injection; the tumors from OT-I cells group were relapsed tumors collected 32 days after OT-I cell injection. The numbers on the bottom represent relative expression levels of gp100 and OVA normalized against GAPGH. **E** and **F,** Survival plots for survivors from combination treatment after being first rechallenged with B16-OVA (D; naïve: *n* = 4; survivor: *n* = 6) and then CMT64 (E; naïve: *n* = 5; survivor: *n* = 6) cells. RGDOX: Delta-24-RGDOX. ns, not significant (*P* > 0.05); *, *P* < 0.05; **, *P* < 0.01. log-rank test.

We have reported previously that localized intratumoral injections of Delta-24-RGDOX induce efficacious systemic antitumor immunity (15). Because intratumorally injected TAA-targeting T cells displayed tumor tropism, to take advantage of the complementary properties of adoptive T-cell therapy and oncolytic virotherapy, we combined these two agents to treat mice with disseminated melanomas derived from B16-OVA cells (Supplementary Fig. S1; ref. 15). We started the treatment with the intratumoral injection of OT-I T cells (OVA-specific CTLs isolated from OT-I mice; Supplementary Fig. S2B) first followed by three doses of Delta-24-RGDOX (Fig. 2A). The first dose of virus was delivered on day 11, instead of day 7, after tumor implantation when the tumors were larger than those in our previous study (Fig. 2A; ref. 15). We found that the combination of the T cells and Delta-24-RGDOX was more potent in inhibiting the growth of treated and untreated disseminated tumors (Fig. 2B) and resulted in an improved survival rate compared with the rate achieved with either agent alone (Fig. 2C). Compared with the PBS control group, OT-I T cells alone significantly increased the median survival time (Fig. 2C), while CTLs from wild-type C57BL/6 mice showed no therapeutic benefit (Supplementary Fig. S3). Interestingly, we observed relapse of the regressed tumors in the group treated with T cells alone (Fig. 2B), which recapitulated the effect of CAR T-cell therapy in patients with cancer (5). Furthermore, assessment of the levels of TAAs in the tumors revealed that OVA protein, the target of adoptive T-cell therapy, was remarkably downregulated in the relapsed tumors from T cell–treated group compared with the ones from PBS or Delta-24-RGDOX–treated groups (Fig. 2D), which is consistent with antigen escape of relapsed cancers observed during CAR T-cell therapy (5). Nevertheless, tumor relapse was not observed in most of the mice from the combination group (Fig. 2B), indicating that the combination induces more sustainable tumor regression than T cells alone. Because we started viral treatment later but used the same viral dose as in a previous study (15), we did not observe the therapeutic benefit of virotherapy alone seen in our previous study (Fig. 2A–C). In addition, the survivors from the combination group were resistant to rechallenging with B16-OVA cells but were susceptible to mouse lung carcinoma CMT64 cells, indicating the development of specific immune memory against the treated tumor cells (Fig. 2E and F). Overall, the combination of TAA-targeting T cells and Delta-24-RGDOX mediates sustainable systemic antitumor activity and immune memory, while adoptive T-cell therapy alone suffers from tumor relapse, and Delta-24-RGDOX alone shows no therapeutic benefit in experimental settings with large tumors.

### Delta-24-RGDOX Enhances a Systemic Inflammatory Response During Localized Intratumoral Treatment with TAA-targeting T Cells

**FIGURE 3:**
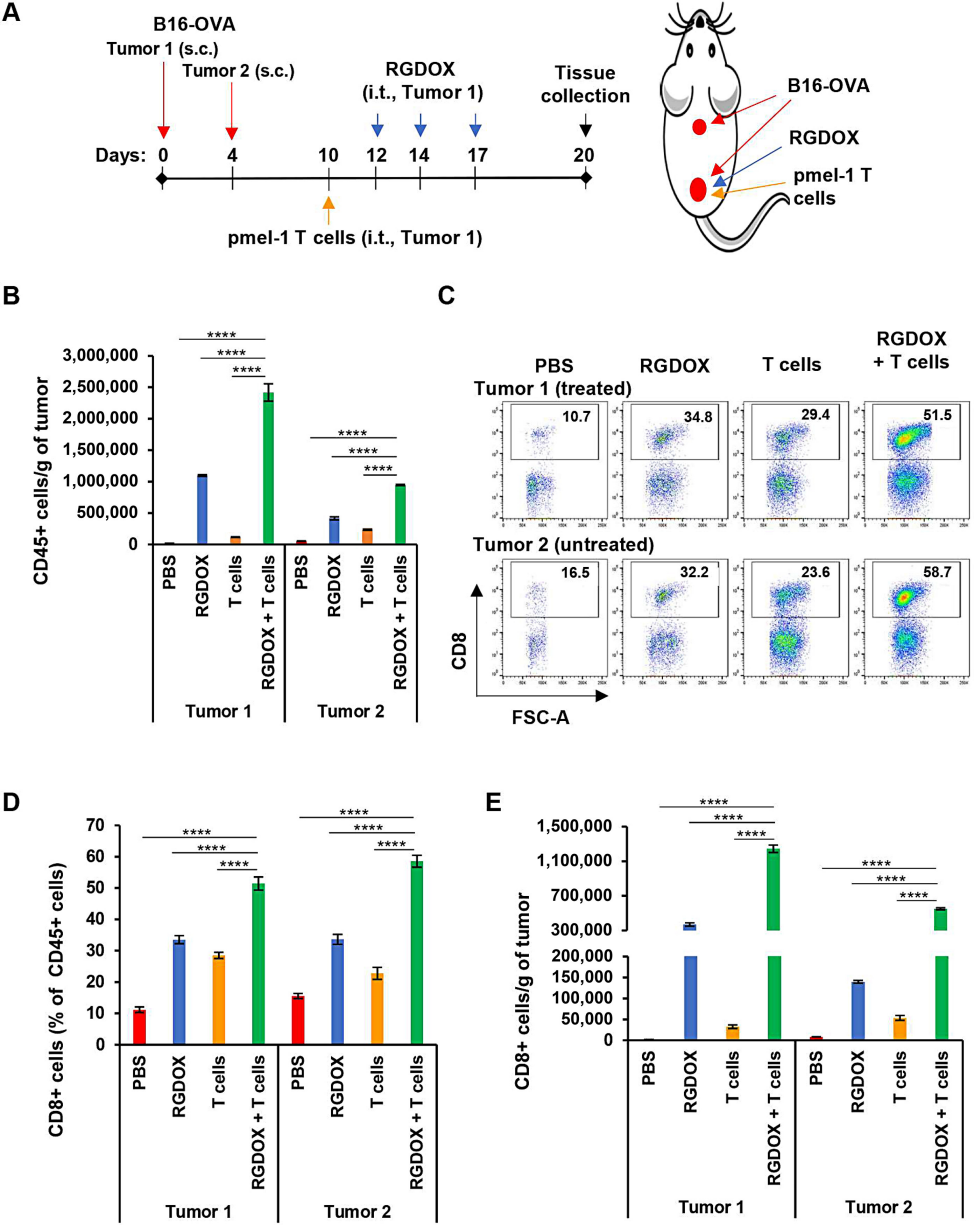
Increased inflammation in treated (Tumor 1) and untreated distant (Tumor 2) tumors induced by combining TAA-specific T cells and Delta-24-RGDOX. **A,** A cartoon depiction of the treatment scheme for schedule (left) and positions of tumor implantation, T-cell and virus injection (right). s.c.: subcutaneously; i.t.: intratumorally. Leukocytes from the tumors in the treatment groups were profiled with flow cytometry. **B,** Quantification of leukocyte (CD45^+^ cells) density in the tumors from each treatment group. Representative flow cytometry dot plots (**C**) and their quantification (**D**) to show CD8^+^ leukocyte frequencies in the indicated treatment groups. **E,** Quantification of CD8^+^ leukocyte density in the tumors from each treatment group. Leukocytes were from grouped tissue (4 to 7 mice/group) and processed in triplicate. The numbers in the upright corner of the dot plots in C indicate the frequency of CD8^+^ cells (percent of leukocytes). The columns in the graphs represent the mean ± SD (*n* = 3). RGDOX: Delta-24-RGDOX; T cells: pmel-1 T cells; ****, *P* < 0.0001. Two-tailed Student *t* test.

We speculated that intratumoral injections of Delta-24-RGDOX after TAA-targeting T cells would enhance the inflammatory response in both treated and untreated tumors, leading to enhanced antitumor immunity. To facilitate the study, we used pmel-1 T cells to treat B16-OVA tumors. Therefore, we could track adopted T cells with Thy1.1 and endogenous OVA-specific CTLs with tetramer later in the study. In a s.c./s.c. B16F10-RFLuc-3 tumor model (Supplementary Fig. S4A), combination of pmel-1 T cells and the virus was more potent to inhibit the growth of both treated and untreated tumors than either of the agents by day 20 (Supplementary Fig. S4B) although the combination did not improve the survival rate significantly by day 50 (Supplementary Fig. S4C). Thus, in a similar experimental setting as described above (Fig. 3A), we collected the tumors on day 20 to profile the tumor-infiltrating lymphocytes. We found the virus synergistically increased the density of leukocytes (CD45^+^) cells in both tumors, although to a lesser extent in the untreated tumors (Fig. 3B; CDI: 0.36 in Tumor 1; 0.50 in Tumor 2). Among these cells, the virus augmented the CD8^+^ cell frequency (Fig. 3C and D), and the combination synergistically increased the CD8^+^ cell density in both tumors (Fig. 3E; CDI: 0.47 in Tumor 1; 0.26 in Tumor 2). The CD8 coreceptor is predominantly expressed on the surface of CTLs. This receptor may also be expressed by some natural killer (NK) or dendritic cells (DC) in the tumor milieu (25, 26). Upregulation of the three cell populations is correlated with a proinflammatory response to clear viral infections and/or eliminate cancer cells (14, 15, 25–27). According to our previous experience with the mouse B16 melanoma models, 90% of CD8^+^ leukocytes in tumors from untreated mice and 99% of those from virus-treated mice are CTLs (Supplementary Fig. S5) that can function as the ultimate effector to clear viral infections and eliminate cancer cells. Therefore, this combination strategy synergistically increases the inflammatory response not only in the treated but also in untreated distant tumors, suggesting an abscopal effect of localized intratumoral treatment.

### Localized Treatment with Delta-24-RGDOX Systemically Downregulates Systemic Immune Suppression of Endogenous TAA-specific CTLs

**FIGURE 4:**
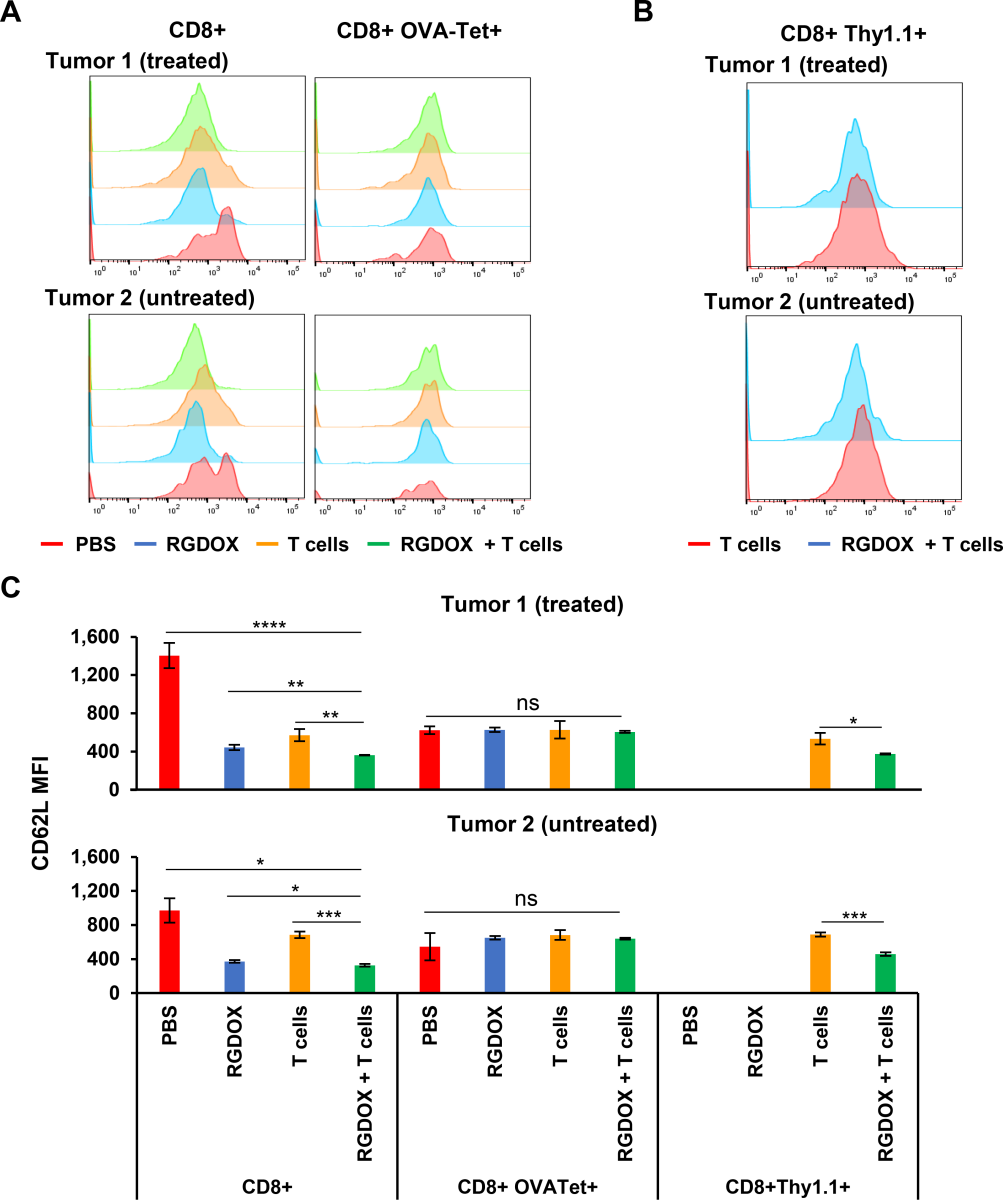
CD62 L downregulation in CD8^+^ leukocytes from treated (Tumor 1) and untreated (Tumor 2) subcutaneous tumors caused by TAA-specific T cells and/or Delta-24-RGDOX. Leukocytes from the tumors in the treatment groups, as depicted in Fig. 3A, were profiled with flow cytometry. Representative flow cytometry histograms showing CD62 L expression on CD8^+^ leukocytes (**A**, left), endogenous OVA-specific CTLs (**A**, right, CD8^+^ OVA-Tet+) and pmel-1 T cells (**B**) from tumors of the indicated treatment groups. **C,** Quantification of the mean fluorescence intensity (MFI) of CD62 L on the cells mentioned in A and B. Leukocytes were from grouped tissue (4 to 7 mice/group) and processed in triplicate. The columns in the graphs represent the mean ± SD (*n* = 3). RGDOX: Delta-24-RGDOX; T cells: pmel-1 T cells. ns, not significant (*P* > 0.05); *, *P* < 0.05; **, *P* < 0.01; ***, *P* < 0.001; ****, *P* < 0.0001. Two-tailed Student *t* test.

**FIGURE 5:**
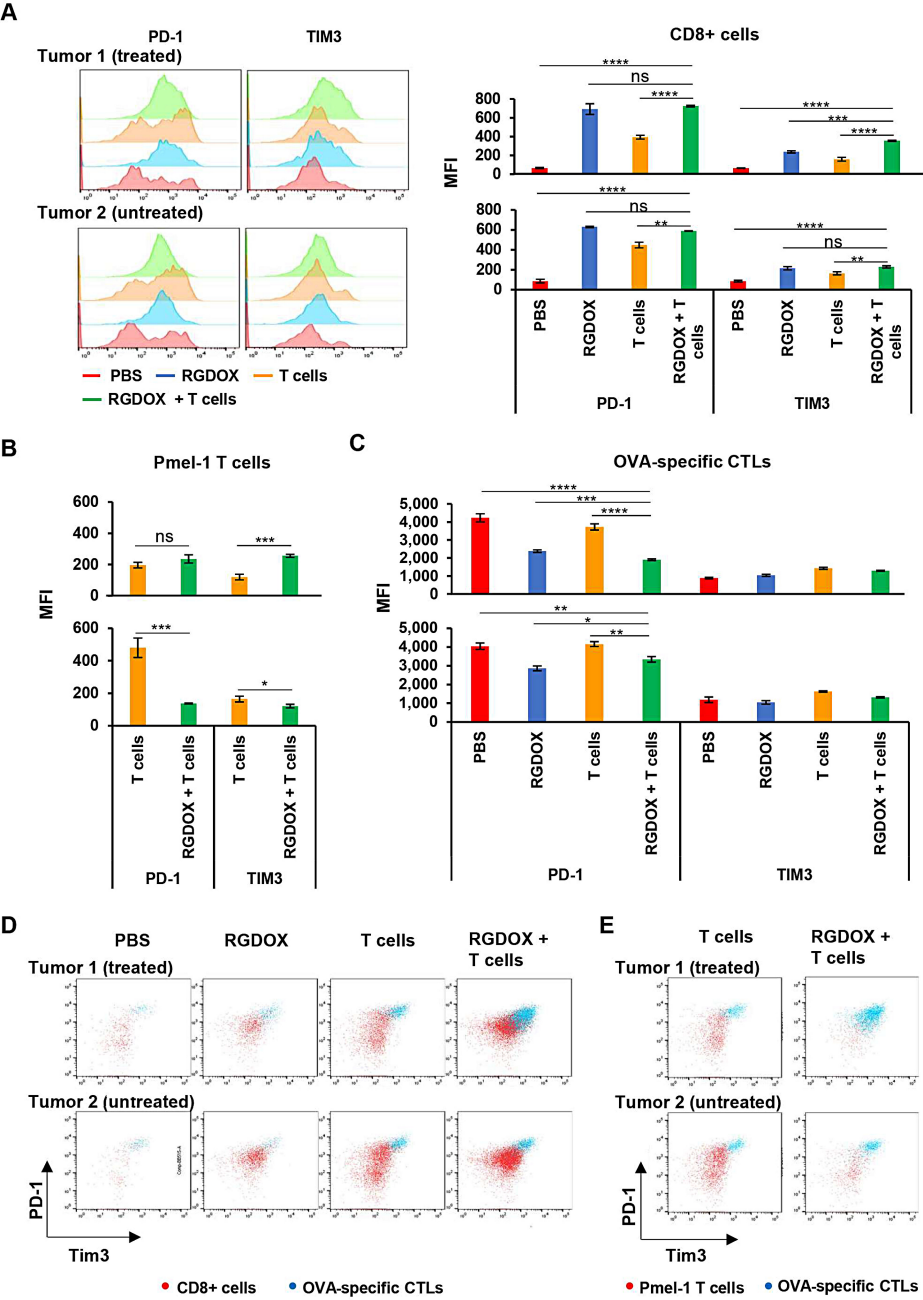
Release of immune suppression of endogenous TAA-specific CTLs from treated (Tumor 1) and untreated (Tumor 2) subcutaneous tumors mediated by Delta-24-RGDOX. PD-1 and TIM3 levels were assessed in CD8^+^ leukocytes (**A**), adoptive pmel-1 T cells (**B**) or endogenous OVA-targeting CTLs (**C**) from the indicated tumors. Top: Tumor 1; bottom: Tumor 2. **A,** Left: Representative flow cytometry histograms; Right: Quantification of MFI of PD-1 or TIM3. **B** and **C,** Quantification of MFI of PD-1 or TIM3. **D** and **E,** Representative flow cytometry dot plots to show relative amount of cell numbers and immune checkpoint protein (PD-1 and TIM3) expression levels between endogenous OVA-specific CTLs and CD8^+^ leukocytes (D) or OVA-specific CTLs and adoptive pmel-1 T cells (E) from the indicated tumors. Leukocytes from the tumors in the treatment groups as depicted in Fig. 3A were profiled with flow cytometry. Leukocytes were from grouped tissue (4 to 7 mice/group) and processed in triplicate. The columns in the graphs represent the mean ± SD (*n* = 3). RGDOX: Delta-24-RGDOX; T cells: pmel-1 T cells. ns, not significant (*P* > 0.05); **, *P* < 0.01; ***, *P* < 0.001; ****, *P* < 0.0001. Two-tailed Student *t* test.

To evaluate the immune status in the tumors, we characterized the immune activation and suppression markers in the following three cell populations: CD8^+^ leukocytes (Fig. 3C), adoptive pmel-1 T cells (Supplementary Fig. S6A), and endogenous OVA-specific CTLs (Supplementary Fig. S6B). First, we compared the expression of CD62 L (L-selectin), an activation marker and a key regulator of T-cell trafficking (28–30), among these cells. Interestingly, compared with CD8^+^ leukocytes from the tumors in the group mock treated with PBS, its expression was maintained at significantly lower levels in all the other examined cells (Fig. 4). The virus had little effect on OVA-specific CTLs, although it downregulated CD62 L expression among CD8^+^ leukocytes and pmel-1 T cells (Fig. 4C), indicating that endogenous tumor-specific CTLs had experienced TCR-mediated activation accompanied by CD62 L ectodomain shedding from their cell surface (29). When we examined the expression of the immune checkpoint proteins PD-1 and TIM3 (T cell immunoglobulin and mucin domain-containing protein 3) on the three cell populations, we found that the virus dramatically increased PD-1 expression levels in CD8^+^ leukocytes from both treated and untreated tumors, while adoptive T cells increased PD-1 levels to a lesser extent (Fig. 5A). Meanwhile, although TIM3 expression changes were not as dramatic as PD-1 in CD8^+^ leukocytes, its expression was modulated by the treatment in the same manner as PD-1 (Fig. 5A). Interestingly, the scenario differed in tumor-targeting adoptive pmel-1 T cells and endogenous OVA-specific CTLs (Fig. 5B and C). For pmel-1 T cells from the treated tumors, the expression levels of the inhibitors were relatively lower than those in CD8^+^ leukocytes, and the virus increased their expression (Fig. 5B, top). For pmel-1 T cells from the untreated tumors, the expression levels of the inhibitors in the T cell–treated group were relatively higher than those in the cells from the treated tumors, and the virus downregulated their expression (Fig. 5B, bottom). However, compared with the previous two cell populations, the expression levels of the inhibitors were remarkably higher in endogenous OVA-specific CTLs (Fig. 5C–E). Importantly, the virus significantly downregulated PD-1 levels in the cells from both treated and untreated tumors, although it did not affect TIM expression to the same extent (Fig. 5C). Overall, during localized intratumoral combination with tumor-targeting T cells, Delta-24-RGDOX downregulates the expression of immune checkpoint proteins on endogenous TAA-specific CTLs in both tumors, although their expression is upregulated on CD8^+^ leukocytes.

### Delta-24-RGDOX Promotes Antigen Spread During Localized Intratumoral Combination with TAA-targeting T Cells

**FIGURE 6:**
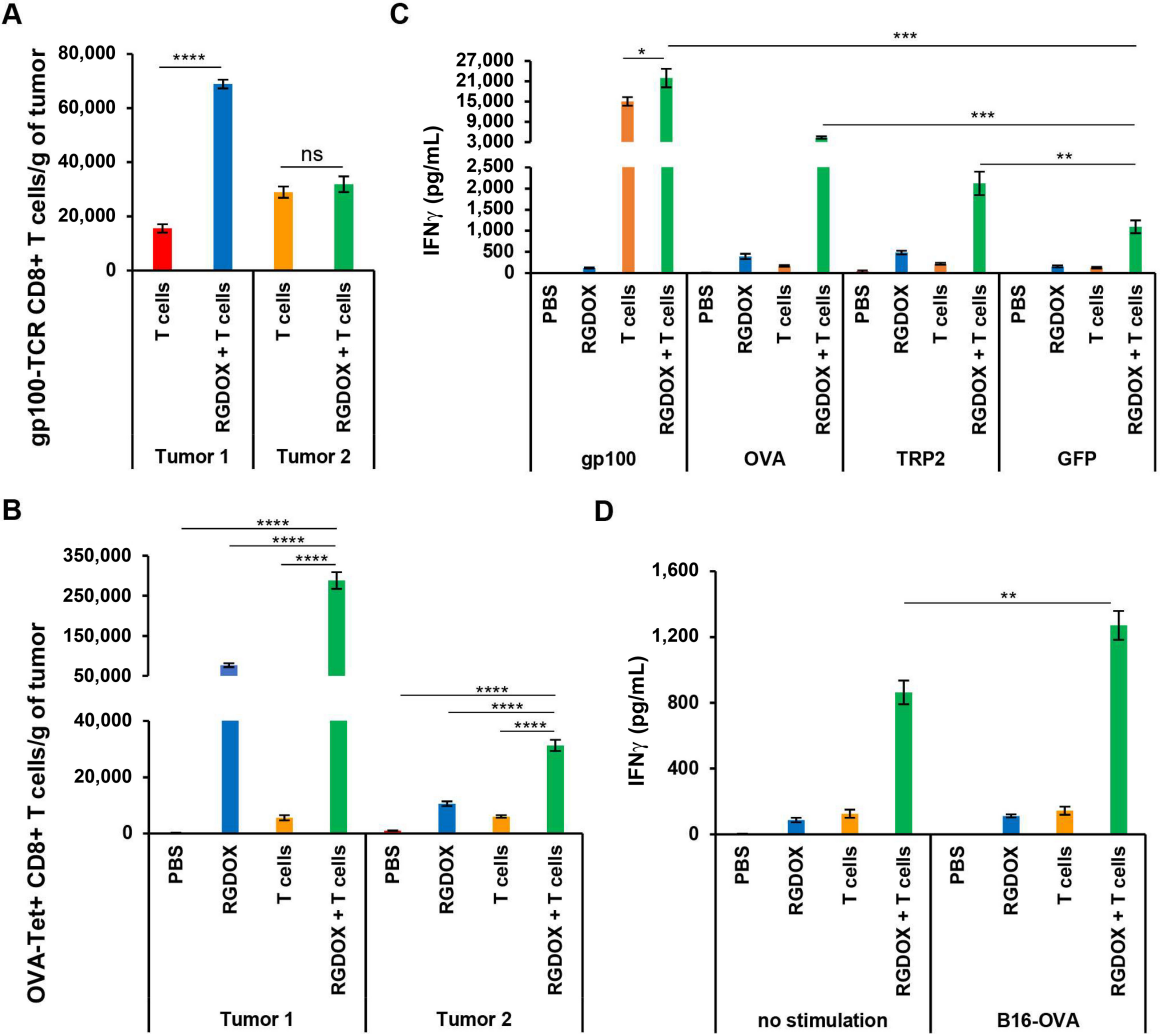
Antigen spread enhanced by combining TAA-specific CTLs and Delta-24-RGDOX in localized intratumoral treatment. Flow cytometry to quantify the density of adoptive TAA-specific pmel-1 T cells (**A**) or endogenous TAA (OVA)-specific CTLs (**B**) in treated (Tumor 1) and untreated (Tumor 2) subcutaneous tumors from the indicated treatment groups. Leukocytes were from grouped tissue (4 to 7 mice/group) and processed in triplicate. ELISA assay to show the amount of IFNγ secreted by the splenocytes from the treatment groups stimulated by epitope peptides of TAAs (OVA, gp100, TRP2) and GFP (**C**), or B16-OVA cells (**D**). Leukocytes or splenocytes were from grouped tissue (4 to 7 mice/group) and processed in triplicate. The columns in the graphs represent the mean ± SD (*n* = 3). RGDOX: Delta-24-RGDOX; T cells: pmel-1 T cells. *, *P* < 0.05; **, *P* < 0.01; ***, *P* < 0.001; ****, *P* < 0.0001. Two-tailed Student *t* test.

To determine whether Delta-24-RGDOX mediates antigen spread, we assessed first the amount of adoptive pmel-1 T cells and endogenous OVA-specific CTLs in the tumors. We found that Delta-24-RGDOX greatly increased the density of pmel-1 T cells in the treated tumors and maintained their density at comparable levels in the untreated tumors although it decreased the frequency of the cell population in CD8^+^ leukocytes (Fig. 6A; Supplementary Fig. S6A). Meanwhile, the virus dramatically increased the density of OVA-specific CTLs in both tumors (Fig. 6B). Moreover, the combination synergistically increased the density of this cell population in both tumors (Fig. 6B; CDI: 0.17 in Tumor 1; 0.50 in Tumor 2). Next, to evaluate the adoptive antitumor immunity induced by the treatments, we stimulated the splenocytes from the mice with epitope peptides of gp100 (the target of adoptive pmel-1 T cells), the other TAAs in B16-OVA cells (OVA and TRP2) and irrelevant GFP. Assessment of IFNγ concentration in the culture medium to evaluate the cell-mediated adoptive immune response against these epitope peptides revealed that, compared with the activity against GFP, the combination significantly augmented the specific activity against OVA and TRP2 while slightly increasing the activity against gp100 (Fig. 6C). Consequently, the combination therapy significantly enhanced the activity of splenocytes against B16-OVA cells expressing these TAAs (Fig. 6D), including gp100 and OVA (Supplementary Fig. S1; ref. 31). The IFNγ in the coculture was produced by DC, NK cells, and peptide-stimulated T cells. Because the virus activated DCs and NK cells in the treated animals, it increased the baseline level of IFNγ in the medium. And it seems there was a synergy between the two treatments for the effect. Collectively, during localized intratumoral combination with TAA-specific T cells, Delta-24-RGDOX augmented antitumor immunity through antigen spread.

## Discussion

Tumor relapse due to antigen escape is an essential problem in adoptive T-cell therapy with limited antigen targets, such as CAR T-cell therapy (17, 32, 34). This issue is especially true for advanced solid tumors because they are more heterogeneous than hematological malignancies (35). Moreover, systemically delivered T cells infiltrate poorly into solid tumors and are susceptible to the immune-suppressive TME (“cold tumor”; ref. 35). On the other hand, OVs, such as the OA Delta-24-RGDOX, can turn “cold tumors” into immune-active “hot tumors” and are expected to induce immune responses against a variety of TAAs (14, 15, 20, 21). Given the complementary properties of these two approaches, we examined the effect of combining the two therapies in immunocompetent mice with disseminated s.c./s.c. tumors. We demonstrated that localized intratumoral delivery of TAA-specific T cells followed by Delta-24-RGDOX sustains adoptive T cell–mediated systemic tumor regression and significantly improves the long-term survival rate in treated mice. Further analysis revealed that Delta-24-RGDOX could potentiate the effect by systemically activating TME and upregulating endogenous CTLs targeting TAAs other than the target of adoptive T cells, a process referred to as, antigen spread, to overcome tumor relapse.

Preclinical and clinical studies indicate that regional and intratumor delivery of CAR T cells is well tolerated and induces regression of solid tumors (6, 7, 24, 36–40). Compared to intravenous delivery of GD2 CAR T cells, in patients with clinical response, intracerebroventricular injection of the cells elicits significantly higher levels of inflammatory cytokines in cerebrospinal fluid, while their levels are relatively lower in blood, suggesting greater efficacy at the tumor site and less systemic toxicity (7). In mice with peritoneal carcinomatosis, regional intraperitoneal infusion of CAR T cells results in superior protection against carcinoembryonic antigen (CEA+) peritoneal tumors than systemically infused CAR T cells (40). Here, we show TAA-specific T cells injected intratumorally in one subcutaneous tumor have tumor tropism for distant subcutaneous and intracranial tumors and TDLNs (Fig. 1). Consistently, we found that localized intratumoral injection of TAA-specific T cells significantly induced regression in both treated and untreated distant tumors and improved the survival rate (Fig. 2B and C). The dose of OT-I T cells used in this study was only one-tenth of that used to treat a single subcutaneous B16-OVA melanoma through tail vein delivery, as reported previously (41), and the intratumoral approach shows comparable efficacy to prolong medium survival time (ref. 41; Fig. 2C). Because of a lack of available tumor-specific antigens, CAR T cell–targeted TAAs may be shared by normal tissue, resulting in stronger toxicities versus conventional tumor-infiltrating lymphocytes because of their clonal specificity toward a single antigen (42). Unlike intravenous delivery, intratumoral T-cell delivery is expected to limit this type of toxicity directly from T cells more locally. Moreover, this approach is expected to cause less toxicity from systemic cytokine release syndrome. In addition, because locoregional delivery of CAR T cells is potent enough to induce tumor regression even without lymphodepletion (7), this localized strategy is promising to avoid the toxicity from lymphodepleting chemotherapy as well.

The activity of tumor-infiltrating T cells is determined by stimulating signals in the TME. T cells recognize stimuli through cell surface receptors that trigger downstream responses. Analyzing these cell surface molecules provides clues about the experience and activity status of T cells in the tumor. For example, when the cell is activated, CD62 L is cleaved at K283-S284, followed by its ectodomain shedding from the cell surface (43). CD62 L shedding from antigen-activated T cells is required for the acquisition of lytic activity following antigen recognition (44) and prevents their reentry into peripheral lymph nodes (29). Thus, the downregulation of CD62 L is an indicator of the activation of T cells. In our experimental setting, the CD62 L level on CD8^+^ leukocytes from the PBS control group was significantly higher than that on CD8^+^ leukocytes from the other treatment groups, endogenous OVA-specific CTLs and adoptive pmel-1 T cells from all groups (Fig. 4). This result indicates that, except for CD8^+^ leukocytes from the PBS control, all the cell populations from the treatment groups experienced activation (Fig. 4). After Delta-24-RGDOX treatment, the virus effectively activated and expanded naïve T cells specific for viral antigens in virus-treated groups, resulted in low CD62 L level in these cells. The rest of the low CD62L-expressing cells are activated adoptive T cells and reactivated resident-memory T cells, including endogenous tumor-specific T cells that are recruited to and expanded at the tumor sites. Because endogenous TAA-specific CTLs and adoptive pmel-1 T cells display significantly lower levels of CD62 L in tumors despite the treatment, it is reasonable to speculate that the downregulation of CD62 L on CD8^+^ leukocytes that correlated with viral injections is mainly due to the activation and expansion of CTLs specific for viral antigens (Figs. 3 and 4; Supplementary Fig. S5).

The immune checkpoint is essential for regulating T-cell activity (45). Among the immune checkpoint proteins, PD-1 and TIM3 suppress T cells later in an immune response, primarily in antigen-expressing organs or tumors (46, 47). These proteins are overexpressed on dysfunctional or “exhausted” T cells in chronic viral infections and cancer (46, 47). They are targets for immune checkpoint blockade in cancer immunotherapy (46–48). Compared with TIM3, Delta-24-RGDOX upregulates the expression of PD-1 on CD8^+^ leukocytes much more dramatically (Fig. 5A). This is consistent with our observation of Delta-24-RGDOX-mediated PD-1 upregulation in tumor-infiltrating CTLs using the same melanoma mouse model (15). Upon TCR activation, PD-1 expression on naïve T cells is induced (49). This transient expression decreases in the absence of TCR signaling but is maintained upon chronic activation with a persisting epitope target to keep the T-cell activity in check (50). On the other hand, the adoptive T cells were less responsive to the upregulation of PD-1 by the virus than CD8^+^ leukocytes (Fig. 5A and B). The expression of the inhibitors on the pmel-1 T cells injected into the first tumor remained at steady low levels and was increased by Delta-24-RGDOX injected into the same tumor (Fig. 5B, top), indicating that the cells initiated negative feedback after further activation by the virus in this tumor. However, the pmel-1 T cells in the untreated tumors had higher levels of the checkpoint proteins, suggesting that the cells were more inhibited by the relatively suppressive TME when they migrated to the second tumor (Fig. 5B, bottom). However, the inhibition was reversed by Delta-24-RGDOX (Fig. 5B, bottom). Unlike adoptive pmel-1 T cells, endogenous OVA-specific CTLs were highly repressed in tumors with PD-1 levels more than 10 times higher than those on pmel-1 T cells (Fig. 5B and C). Their PD-1 expression was significantly downregulated by the virus in both tumors (Fig. 5C). It indicates the release of immune suppression, leading to the expansion of this cell population and a significant increase in its density in the tumors (Fig. 6B). Given the opposite effect of the virus on CD8^+^ leukocytes, which include viral antigen-specific CTLs, these findings suggest a transition from antiviral immunity to antitumor immunity at this timepoint.

Because of clonal selection pressure favoring tumor cells lacking the targeted antigens, cancers treated with T cells targeting limited TAAs can undergo antigen escape (Fig. 2D; ref. 5). This problem with CAR T-cell therapy can be partially addressed by developing CAR T cells targeting multiple TAAs. However, as mentioned above, to date, effective available targets are limited to treating a variety of solid tumors (5). Furthermore, CAR T-cell therapy involves *ex vivo* immune cell manipulation that is both expensive and labor intensive (18, 51), which limits its availability to patients with cancer. As we demonstrate, Delta-24-RGDOX increases the density and activity of endogenous tumor-targeting CTLs during adoptive T-cell therapy while maintaining that of adoptive T cells (Fig. 6), leading to sustainable systemic tumor regression to prevent cancer relapse (Fig. 2B and C). According to our previous study, Delta-24-RGDOX is more potent than its predecessor, Delta-24-RGD, to directly stimulate the activity and proliferation of TAA-specific T cells through OX40 L expression on the infected tumor cells (14). This indicates that with lower doses and limited types of adoptive tumor-targeting T cells, Delta-24-RGDOX can potentiate the therapeutic effect of these cells by upregulating the CTL repertoire against other TAAs to reduce the chance of cancer relapse.

Because CARs against mouse TAAs are rarely available, most studies on combination therapy with OVs and CAR T cells have been performed in NSG mice with virotherapy followed by human CAR T cells (18). Although the system can assess the activity of CAR T cells and OVs against human xenografts in mice, it cannot recapitulate all the interplay between the two agents, other immune cells and tumor cells. In this study, we combined TAA-specific T cells with Delta-24-RGDOX to treat mice with disseminated syngeneic tumors. This therapeutic model includes the effects of the interactions between all the factors mentioned above. In addition, in contrast to previous studies on treatments that began with OV administration followed by intravenously delivered CAR T cells in mouse tumor models with a single tumor to attract adoptive T cells to the tumor (18), we injected TAA-targeting T cells directly into the first tumor followed by Delta-24-RGDOX to evaluate the abscopal effect of the therapy. Although a few studies have used mouse CAR T cells against mouse CD19 or human EGFRvIII combined with OVs in syngeneic mouse models, either the mice were irradiated for lymphodepletion, or the CAR T cells were delivered intravenously (52–54). Furthermore, the adenovirus is a potent immune stimulator to induce innate and adaptive immunity against the virus. Although the virus-mediated TME activation favors the activity of the adoptive T cells, administering the virus earlier may worsen the competition between the antiviral immunity and the adoptive T cells for the immune resource to expand the corresponding T-cell populations. To this end, while Delta-24-RGDOX increased the density and activity of antitumor T cells (Fig. 6), it decreased frequency of the adoptive T cells (Supplementary Fig. S6A), indicating the expansion of antiviral T-cell populations. In the B16-OVA-OT-I therapy model, we observed efficacious antitumor effect by OT-I T cells alone albeit the effect was not sustainable (Fig. 2B). Thus, adjuvant treatment with the virus after effective ACT postponed the immune resource competition to give more time for OT-I cells to exert their antitumor activity. Virus-mediated effect kicked in later to sustain OT-I cell–induced tumor regression. On the other hand, as reported previously (55), we found that pmel-1 T cells were less effective than OT-I T cells to induce regression in B16 melanomas. Because these tumors were aggressive, unlike OT-I T cells, pmel-1 T cells were not potent enough to create a favorable time window for adjuvant treatment with Delta-24-RGDOX to further prolong animal survival (Fig. 2C; Supplementary Fig. S4C). Therefore, the efficacy of ACT may be a critical factor to determine the long-term benefit of this adjuvant adenovirus therapy.

In conclusion, for the first time, by analyzing the immune status of different tumor-infiltrating T-cell populations and the TAA-specific immune response, we demonstrate that Delta-24-RGDOX activates TME, leading to reduction of immune suppression of endogenous TAA-specific CTLs and promotion of antigen spread to potentiate the effect of adoptive TAA-specific T cells, resulted in sustainable systemic tumor regression in localized intratumoral treatment. Our data indicate that OVs are promising candidates for adjuvant therapy to enhance the efficacy of adoptive T-cell therapy with limited targets, such as CAR T-cell therapy, and prevent tumor relapse. Moreover, this approach is expected to reduce toxicity due to systemic cytokine release syndrome or lymphodepleting chemotherapy and cut the cost of *ex vivo* T-cell engineering and expansion. Therefore, our proof-of-principle study here supports translating this strategy into the clinic to benefit more patients with advanced solid tumors.

## Supplementary Material

Supplementary Table 1 Antibody information

Supplementary Figure 1 gp100 and ovalbumin (OVA) expression in indicated mouse melanoma cell lines. Cell lysates were analyzed with immunoblotting. GAPDH levels are shown as a protein loading control.

Supplementary Figure 2 Expression of TCRs in CD8+ T cells from pmel-1 (A, gp100-Tet+) or OT-I (B, OVA-Tet+) transgenic mice. Splenocytes from the mice were stained with tetramers binding with TCR specific for gp100 (pmel-1) or OVA (OT-I), and antibody against CD8. Stained cells were analyzed with flow cytometry. The numbers in upright corner of the dot plots indicate the frequency (% of splenocytes) of gp100-targeting (A) or OVA-targeting (B) in CD8+ T cells.

Supplementary Figure 3 Survival plots of mice with s.c./s.c. melanomas treated with PBS or wildtype (WT) CD8+ T cells injected into the s.c. Tumor 1. PBS: n=8; WT CTLs: n=5. ns: not significant (p > 0.05). log-rank test.

Supplementary Figure 4 The effect of combining pmel-1 T cells and Delta-24-RGDOX. A, A cartoon depiction of the treatment scheme for schedule (left) and positions of tumor implantation, T-cell and virus injection (right). s.c.: subcutaneously; i.t.: intratumorally. B, Plots of the bioluminescence of the tumors from the indicated treatment groups. C, Survival plots of the treatment groups. n = 9 except n = 8 for combination group. RGDOX: Delta-24-RGDOX. ns: not significant (p > 0.05); **: p<0.01. log-rank test.

Supplementary Figure 5 Expression of CD3 and CD8 on CD45+ leukocytes from B16F10-RFLuc-3 tumors. Delta-24-RGDOX was injected into the s.c. tumors on Days 8, 11 and 14. Leukocytes from the tumors were profiled through flow cytometry on Day 18. RGDOX: Delta-24-RGDOX.

Supplementary Figure 6 Frequency of adoptive pmel-1 T-cells (A) or endogenous OVA-specific CTLs (B) in CD8+ leukocytes from treated (Tumor 1) and untreated (Tumor 2) s.c. tumors. Leukocytes from the tumors in the treatment groups as depicted in Fig. 3A were profiled with flow cytometry. Thy1.1+ or OVA-Tet+ CD8+ T-cells were gated as pmel-1 T-cells or OVA-specific CTLs respectively. Shown are represent dot plots of flow cytometry. Leukocytes were from grouped tissue (4 to 7 mice/group) and processed in triplicate. The numbers in the upright corner of the dot plots indicate percentage of CD8+ leukocytes. RGDOX: Delta-24-RGDOX; T cells: pmel-1 T-cells.
