# Supplementary material for: Adjuvant Therapy with Oncolytic Adenovirus Delta-24-RGDOX After Intratumoral Adoptive T-cell Therapy Promotes Antigen Spread to Sustain Systemic Antitumor Immunity
Source: Cancer Res Commun. 2023 Jun 27;3(6):1118–31. doi: 10.1158/2767-9764.CRC-23-0054 (PMC10295804; doi:10.1158/2767-9764.CRC-23-0054)
Supplement: Supplementary Table 1 — Antibody information [file crc-23-0054-s01.docx]

**Supplemental Table 1: Antibody Information**

| **Application** | **Antibody/Tetramer** | **Company** | **Catalog#** | **Dilution** |
| --- | --- | --- | --- | --- |
| Adenoviral titration | Goat anti-Adenovirus | EMD Millopore | AB1056 | 1:500 |
| Adenoviral titration | Rabbit Anti-Goat IgG Antibody (H+L),  Biotinylated | Vector Laboratories | BA-5000 | 1:500 |
| Western blot | Rabbit anti-Melanoma gp100 | abcam | ab137078 | 1:2000 |
| Western blot | Rabbit anti‑Chicken Ovalbumin (OVA) | LifeSpan BioSciences | LS-C153793 | 1:1000 |
| Western blot | Rabbit anti-GAPDH | Santa Cruz Blotechnology | SC-32233 | 1:2000 |
| Western blot | Anti-Rabbit IgG-HRP | Cell Signaling Technology | 7074P2 | 1:5000 |
| Western blot | anti-Mouse IgG-HRP | Invitrogen | SA1-100 | 1:5000 |
| Flow cytometry | CD16/CD32 Monoclonal Antibody (93) | eBioscience | **14-0161-82** | 1:50 |
| Flow cytometry | CD90.1 (Thy1.1) eFluor 450 | eBioscience | 48-0500-82 | 1:200 |
| Flow cytometry | CD8a allophycocyanin (APC) | eBioscience | 17-0081-81 | 1:200 |
| Flow cytometry | PD-1 fluorescein isothiocyanate (FITC) | eBioscience | 11-9981-81 | 1:200 |
| Flow cytometry | TIM3 PE-Cyanine7 | eBioscience | 25-5870-80 | 1:100 |
| Flow cytometry | CD62L APC-eFlour 780 | eBioscience | 47-0621-80 | 1:200 |
| Flow cytometry | CD45 BV786 | BD Biosciences | 564225 | 1:100 |
| Flow cytometry | Tetramer/PE - H-2 Kb OVA (SIINFEKL) (OVA-Tet) | MHC Tetramer Production Facility, Baylor College of Medicine |  | 1:400 |
| Flow cytometry | Tetramer/PE - H-2Db mgp100 (EGSRNQDWL) (gp100-Tet) | MHC Tetramer Production Facility, Baylor College of Medicine |  | 1:200 |

**References:**
