# Supplementary material for: Adjuvant Therapy with Oncolytic Adenovirus Delta-24-RGDOX After Intratumoral Adoptive T-cell Therapy Promotes Antigen Spread to Sustain Systemic Antitumor Immunity
Source: Cancer Res Commun. 2023 Jun 27;3(6):1118–31. doi: 10.1158/2767-9764.CRC-23-0054 (PMC10295804; doi:10.1158/2767-9764.CRC-23-0054)
Supplement: Supplementary Figure 1 — gp100 and ovalbumin (OVA) expression in indicated mouse melanoma cell lines. Cell lysates were analyzed with immunoblotting. GAPDH levels are shown as a protein loading control. [file crc-23-0054-s02.pptx]

## Slide 1
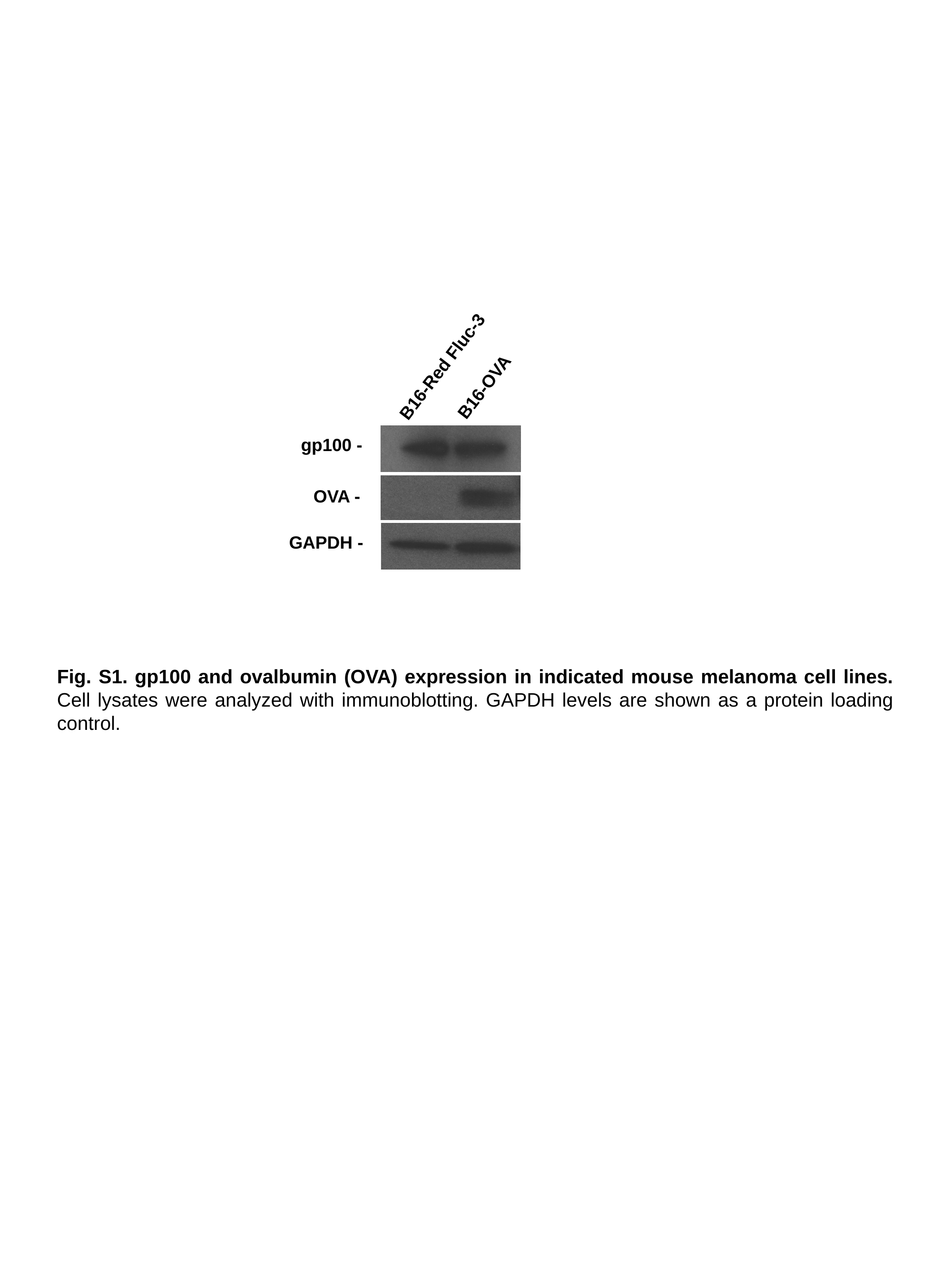

B16-Red Fluc-3
B16-OVA
gp100 -
OVA -
GAPDH -
Fig. S1. gp100 and ovalbumin (OVA) expression in indicated mouse melanoma cell lines. Cell lysates were analyzed with immunoblotting. GAPDH levels are shown as a protein loading control.
