# Supplementary material for: Adjuvant Therapy with Oncolytic Adenovirus Delta-24-RGDOX After Intratumoral Adoptive T-cell Therapy Promotes Antigen Spread to Sustain Systemic Antitumor Immunity
Source: Cancer Res Commun. 2023 Jun 27;3(6):1118–31. doi: 10.1158/2767-9764.CRC-23-0054 (PMC10295804; doi:10.1158/2767-9764.CRC-23-0054)
Supplement: Supplementary Figure 2 — Expression of TCRs in CD8+ T cells from pmel-1 (A, gp100-Tet+) or OT-I (B, OVA-Tet+) transgenic mice. Splenocytes from the mice were stained with tetramers binding with TCR specific for gp100 (pmel-1) or OVA (OT-I), and antibody against CD8. Stained cells were analyzed with flow cytometry. The numbers in upright corner of the dot plots indicate the frequency (% of splenocytes) of gp100-targeting (A) or OVA-targeting (B) in CD8+ T cells. [file crc-23-0054-s03.pptx]

## Slide 1
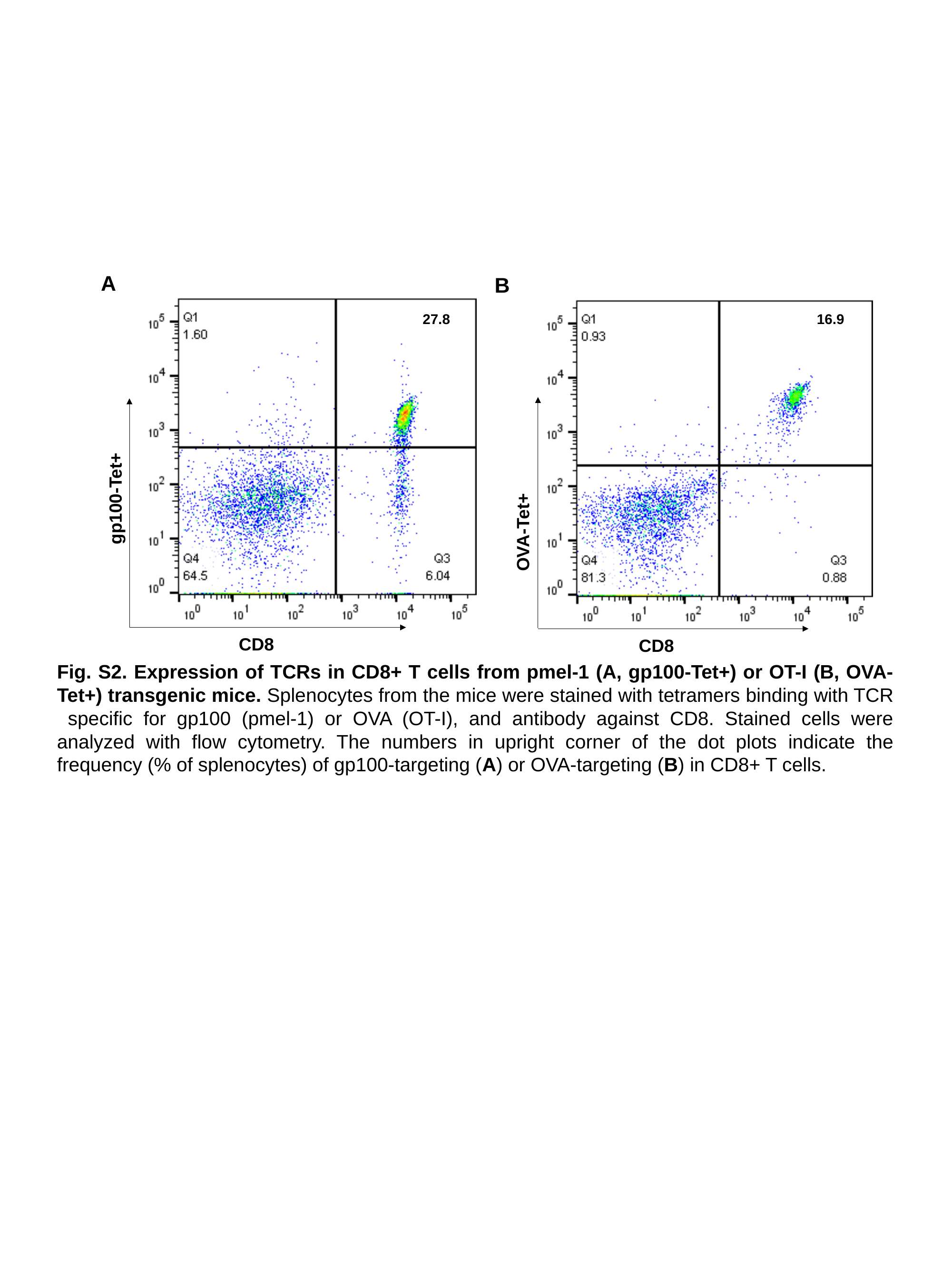

A
B
OVA-Tet+
CD8
27.8
16.9
gp100-Tet+
CD8
Fig. S2. Expression of TCRs in CD8+ T cells from pmel-1 (A, gp100-Tet+) or OT-I (B, OVA-Tet+) transgenic mice. Splenocytes from the mice were stained with tetramers binding with TCR specific for gp100 (pmel-1) or OVA (OT-I), and antibody against CD8. Stained cells were analyzed with flow cytometry. The numbers in upright corner of the dot plots indicate the frequency (% of splenocytes) of gp100-targeting (A) or OVA-targeting (B) in CD8+ T cells.
