# Supplementary material for: Adjuvant Therapy with Oncolytic Adenovirus Delta-24-RGDOX After Intratumoral Adoptive T-cell Therapy Promotes Antigen Spread to Sustain Systemic Antitumor Immunity
Source: Cancer Res Commun. 2023 Jun 27;3(6):1118–31. doi: 10.1158/2767-9764.CRC-23-0054 (PMC10295804; doi:10.1158/2767-9764.CRC-23-0054)
Supplement: Supplementary Figure 3 — Survival plots of mice with s.c./s.c. melanomas treated with PBS or wildtype (WT) CD8+ T cells injected into the s.c. Tumor 1. PBS: n=8; WT CTLs: n=5. ns: not significant (p > 0.05). log-rank test. [file crc-23-0054-s04.pptx]

## Slide 1
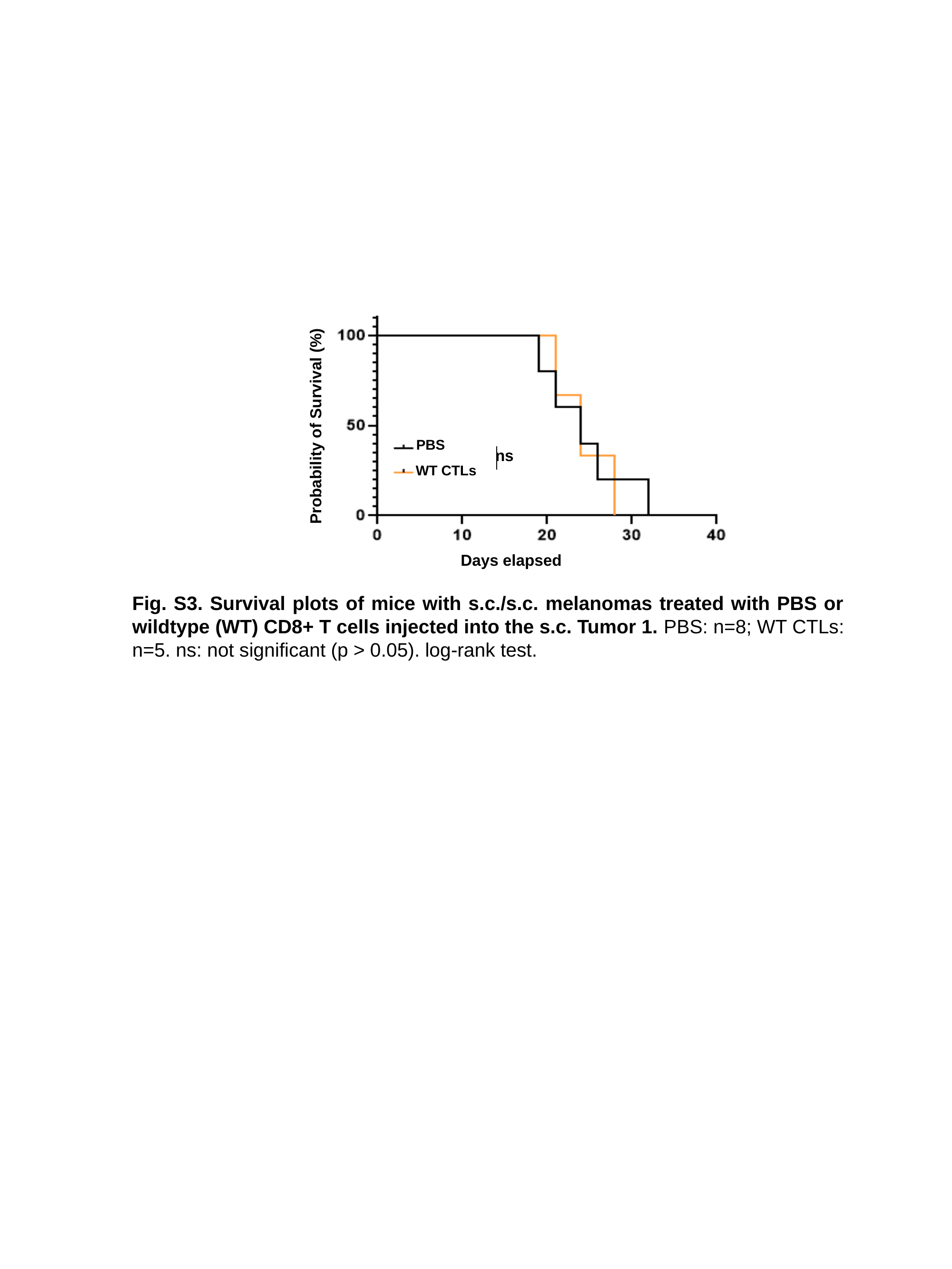

Probability of Survival (%)
PBS
ns
WT CTLs
Days elapsed
Fig. S3. Survival plots of mice with s.c./s.c. melanomas treated with PBS or wildtype (WT) CD8+ T cells injected into the s.c. Tumor 1. PBS: n=8; WT CTLs: n=5. ns: not significant (p > 0.05). log-rank test.
