# Supplementary material for: Adjuvant Therapy with Oncolytic Adenovirus Delta-24-RGDOX After Intratumoral Adoptive T-cell Therapy Promotes Antigen Spread to Sustain Systemic Antitumor Immunity
Source: Cancer Res Commun. 2023 Jun 27;3(6):1118–31. doi: 10.1158/2767-9764.CRC-23-0054 (PMC10295804; doi:10.1158/2767-9764.CRC-23-0054)
Supplement: Supplementary Figure 4 — The effect of combining pmel-1 T cells and Delta-24-RGDOX. A, A cartoon depiction of the treatment scheme for schedule (left) and positions of tumor implantation, T-cell and virus injection (right). s.c.: subcutaneously; i.t.: intratumorally. B, Plots of the bioluminescence of the tumors from the indicated treatment groups. C, Survival plots of the treatment groups. n = 9 except n = 8 for combination group. RGDOX: Delta-24-RGDOX. ns: not significant (p > 0.05); **: p<0.01. log-rank test. [file crc-23-0054-s05.pptx]

## Slide 1
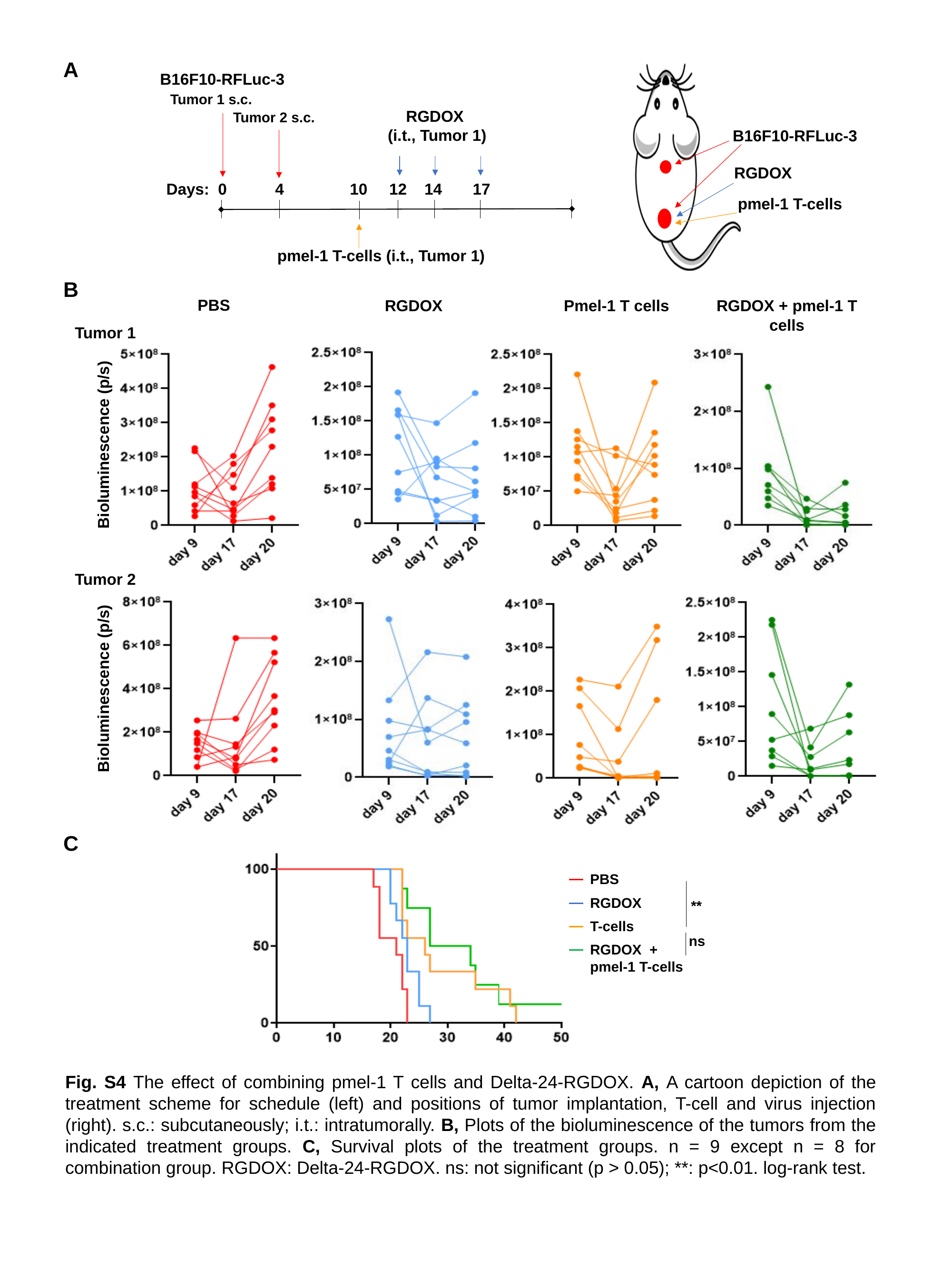

A
B16F10-RFLuc-3
Tumor 1 s.c.
RGDOX
(i.t., Tumor 1)
Tumor 2 s.c.
B16F10-RFLuc-3
RGDOX
Days: 0 4 10 12 14 17
pmel-1 T-cells
pmel-1 T-cells (i.t., Tumor 1)
B
PBS
RGDOX
Pmel-1 T cells
RGDOX + pmel-1 T cells
Tumor 1
Bioluminescence (p/s)
Tumor 2
Bioluminescence (p/s)
C
PBS
RGDOX
**
T-cells
ns
RGDOX + pmel-1 T-cells
Fig. S4 The effect of combining pmel-1 T cells and Delta-24-RGDOX. A, A cartoon depiction of the treatment scheme for schedule (left) and positions of tumor implantation, T-cell and virus injection (right). s.c.: subcutaneously; i.t.: intratumorally. B, Plots of the bioluminescence of the tumors from the indicated treatment groups. C, Survival plots of the treatment groups. n = 9 except n = 8 for combination group. RGDOX: Delta-24-RGDOX. ns: not significant (p > 0.05); **: p<0.01. log-rank test.
