# Supplementary material for: Adjuvant Therapy with Oncolytic Adenovirus Delta-24-RGDOX After Intratumoral Adoptive T-cell Therapy Promotes Antigen Spread to Sustain Systemic Antitumor Immunity
Source: Cancer Res Commun. 2023 Jun 27;3(6):1118–31. doi: 10.1158/2767-9764.CRC-23-0054 (PMC10295804; doi:10.1158/2767-9764.CRC-23-0054)
Supplement: Supplementary Figure 5 — Expression of CD3 and CD8 on CD45+ leukocytes from B16F10-RFLuc-3 tumors. Delta-24-RGDOX was injected into the s.c. tumors on Days 8, 11 and 14. Leukocytes from the tumors were profiled through flow cytometry on Day 18. RGDOX: Delta-24-RGDOX. [file crc-23-0054-s06.pptx]

## Slide 1
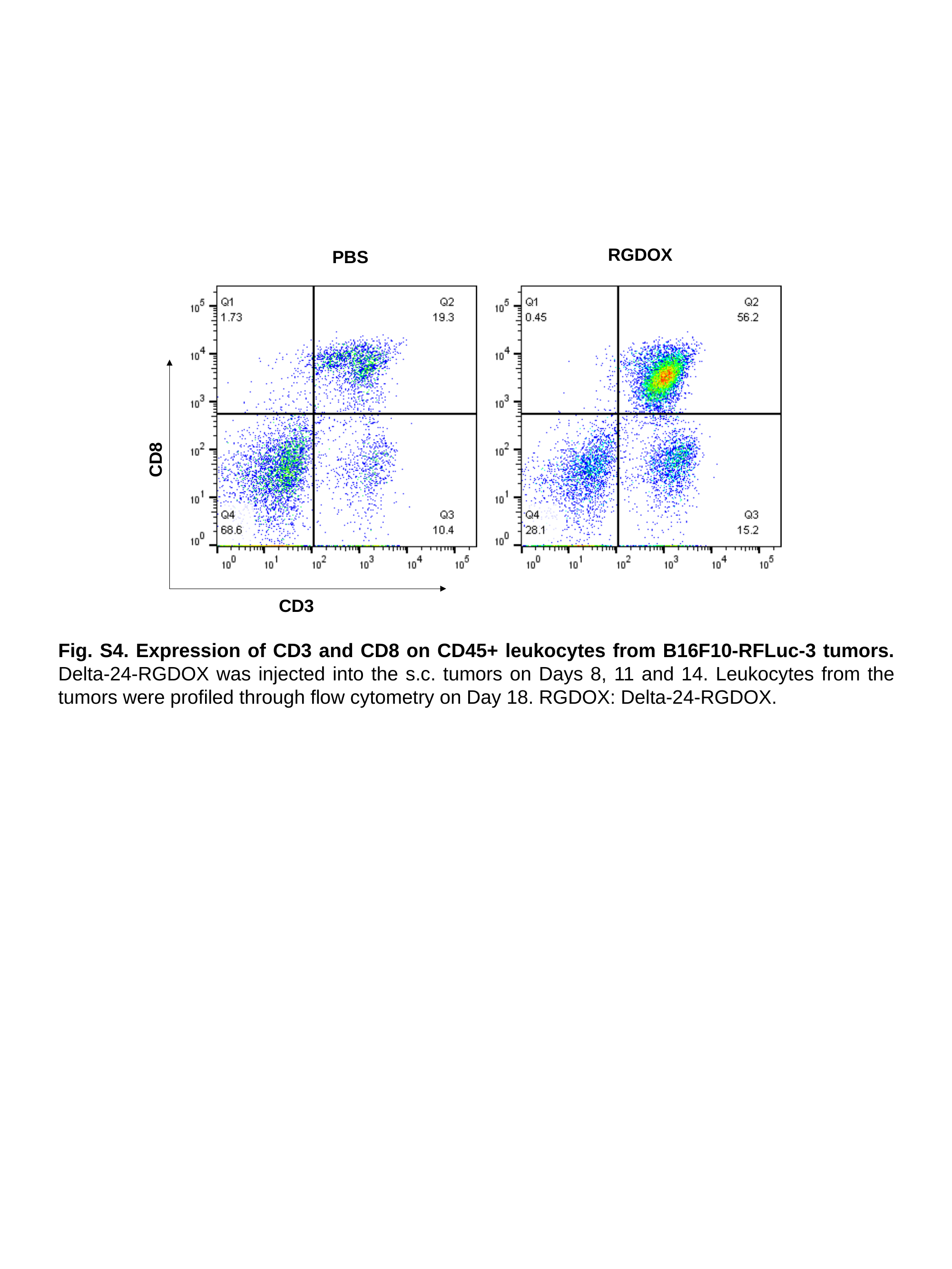

RGDOX
PBS
CD8
CD3
Fig. S4. Expression of CD3 and CD8 on CD45+ leukocytes from B16F10-RFLuc-3 tumors. Delta-24-RGDOX was injected into the s.c. tumors on Days 8, 11 and 14. Leukocytes from the tumors were profiled through flow cytometry on Day 18. RGDOX: Delta-24-RGDOX.
