# Supplementary material for: Adjuvant Therapy with Oncolytic Adenovirus Delta-24-RGDOX After Intratumoral Adoptive T-cell Therapy Promotes Antigen Spread to Sustain Systemic Antitumor Immunity
Source: Cancer Res Commun. 2023 Jun 27;3(6):1118–31. doi: 10.1158/2767-9764.CRC-23-0054 (PMC10295804; doi:10.1158/2767-9764.CRC-23-0054)
Supplement: Supplementary Figure 6 — Frequency of adoptive pmel-1 T-cells (A) or endogenous OVA-specific CTLs (B) in CD8+ leukocytes from treated (Tumor 1) and untreated (Tumor 2) s.c. tumors. Leukocytes from the tumors in the treatment groups as depicted in Fig. 3A were profiled with flow cytometry. Thy1.1+ or OVA-Tet+ CD8+ T-cells were gated as pmel-1 T-cells or OVA-specific CTLs respectively. Shown are represent dot plots of flow cytometry. Leukocytes were from grouped tissue (4 to 7 mice/group) and processed in triplicate. The numbers in the upright corner of the dot plots indicate percentage of CD8+ leukocytes. RGDOX: Delta-24-RGDOX; T cells: pmel-1 T-cells. [file crc-23-0054-s07.pptx]

## Slide 1
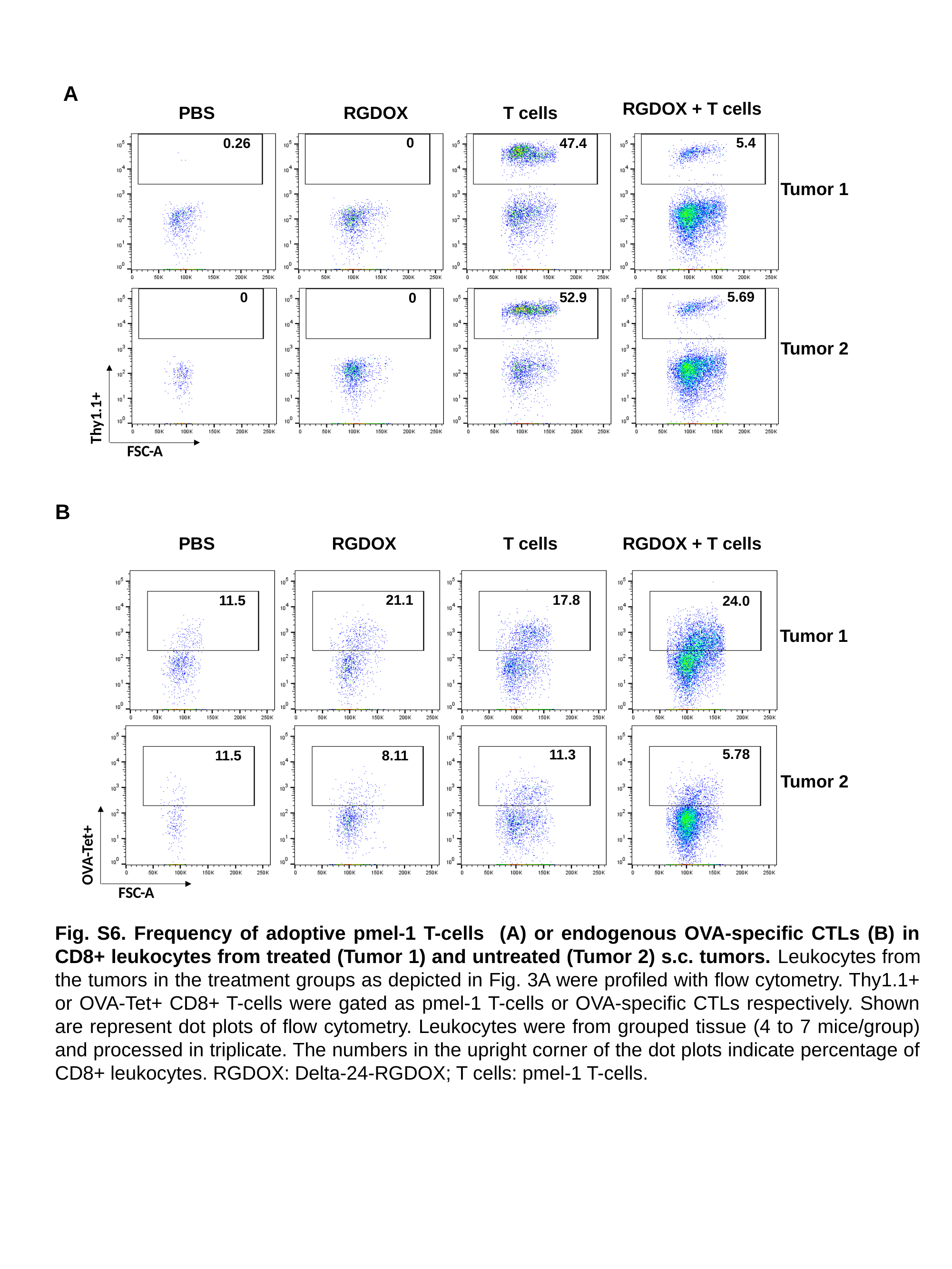

A
RGDOX + T cells
PBS
RGDOX
T cells
0
5.4
0.26
47.4
Tumor 1
5.69
52.9
0
0
Tumor 2
Thy1.1+
FSC-A
B
PBS
RGDOX
T cells
RGDOX + T cells
21.1
17.8
11.5
24.0
Tumor 1
5.78
11.3
8.11
11.5
Tumor 2
OVA-Tet+
FSC-A
Fig. S6. Frequency of adoptive pmel-1 T-cells (A) or endogenous OVA-specific CTLs (B) in CD8+ leukocytes from treated (Tumor 1) and untreated (Tumor 2) s.c. tumors. Leukocytes from the tumors in the treatment groups as depicted in Fig. 3A were profiled with flow cytometry. Thy1.1+ or OVA-Tet+ CD8+ T-cells were gated as pmel-1 T-cells or OVA-specific CTLs respectively. Shown are represent dot plots of flow cytometry. Leukocytes were from grouped tissue (4 to 7 mice/group) and processed in triplicate. The numbers in the upright corner of the dot plots indicate percentage of CD8+ leukocytes. RGDOX: Delta-24-RGDOX; T cells: pmel-1 T-cells.
